# Abundant and diverse *Tetrahymena* species living in the bladder traps of aquatic carnivorous *Utricularia* plants

**DOI:** 10.1038/s41598-019-50123-1

**Published:** 2019-09-20

**Authors:** Chao-Yin Cheng, Shang-Lin Chang, I-Ting Lin, Meng-Chao Yao

**Affiliations:** 1 Institute of Molecular Biology, Academia Sinica, Taipei, Taiwan; 2 Genomics Research Center, Academia Sinica, Taipei, Taiwan

**Keywords:** Microbial communities, Biodiversity

## Abstract

Ciliates are unicellular eukaryotes known for their cellular complexity and wide range of natural habitats. How they adapt to their niches and what roles they play in ecology remain largely unknown. The genus *Tetrahymena* is among the best-studied groups of ciliates and one particular species, *Tetrahymena thermophila*, is a well-known laboratory model organism in cell and molecular biology, making it an excellent candidate for study in protist ecology. Here, based on *cytochrome c oxidase subunit I* (*COX1*) gene barcoding, we identify a total of 19 different putative *Tetrahymena* species and two closely related *Glaucoma* lineages isolated from distinct natural habitats, of which 13 are new species. These latter include 11 *Tetrahymena* species found in the bladder traps of *Utricularia* plants, the most species-rich and widely distributed aquatic carnivorous plant, thus revealing a previously unknown but significant symbiosis of *Tetrahymena* species living among the microbial community of *Utricularia* bladder traps. Additional species were collected using an artificial trap method we have developed. We show that diverse *Tetrahymena* species may live even within the same habitat and that their populations are highly dynamic, suggesting that the diversity and biomass of species worldwide is far greater than currently appreciated.

## Introduction

Ciliated protozoans are unicellular eukaryotic organisms long known for their cellular and behavioral complexity^1^. Several genera (e.g., *Tetrahymena*, *Paramecium*, *Oxytricha*, *Stylonychia Euplotes*, *Blepharisma*, and *Stentor*) have become model taxa for research in many areas of basic biology. *Tetrahymena* is one such genus and *T*. *thermophila* is one of the best-known laboratory eukaryotic models for genetics. It adapts well to growth in axenic proteose-peptone culture and can be easily induced to carry out synchronized sexual conjugation^2,3^. Research driven by the unique biological characteristics of *T*. *thermophila* has contributed to several scientific milestones including the discovery of catalytic RNA (ribozymes) and telomeres and telomerase^4,5^. Recent annotation of its complete genome will facilitate development of even more tools for genomics research^6^.

*Tetrahymena* is typically found in freshwater habitats such as ponds, lakes, rivers, and streams^7–10^. Like most protists, closely related *Tetrahymena* species lacked morphological distinctions and have been difficult to analyze in early studies. DNA barcoding of mitochondrial *cytochrome c oxidase subunit I* (*COX1*) has become a powerful molecular tool for identifying species of *Tetrahymena*^11,12^. The latest study using this tool identified more than 30 new *Tetrahymena* species collected from sites in the U.S.A^13^, and now a total of 81 named species are listed in the NCBI’s GenBank database.

Most known *Tetrahymena* species have been collected from open freshwater habitats and are considered free-living bacterivores. A number of species have been described as being closely associated with a variety of metazoan (primarily invertebrate) hosts such as snails, slugs, mussels, culicine mosquitoes, chironomid larvae, as well as inhabiting the wounds of amphibians or fishes^7,14–17^. The exact relationships between these *Tetrahymena* species and their metazoan hosts have not yet been well described. In addition, the various morphological forms and complex lifecycles of *Tetrahymena* indicate that they possess diverse biological characteristics allowing them to cope with many environmental challenges. For example, some *Tetrahymena* can undergo encystment to survive harsh environments or change their oral morphology (from microstome bacterivore to macrostome carnivore) in response to food availability^1,7,8^. Current knowledge of *Tetrahymena* biology is limited to a few laboratory strains that have been selected for particular traits of interest in scientific research. Topics like how it interacts with metazoan hosts and how it adapts to natural environments requires further study to elucidate. Moreover, even *Tetrahymena* species with highly-similar morphological forms can be divergent for feeding on different bacteria and adapting to different microbial niches. A recent publication reveals the landscape of rapidly evolving genes in *Tetrahymena* and suggests that different *Tetrahymena* species have evolved species-specific genes that may regulate their expression to adapt to environmental changes^18^. Taken together, the basic ecology and natural history of *Tetrahymena* species remains largely unclear.

Recently, one new species, *Tetrahymena utriculariae*, was reported as a specialized bacterivore from the bladder traps of the aquatic carnivorous plant *Utricularia reflexa*^19,20^. The genus *Utricularia* uses its bladder traps to capture and digest small organisms to obtain nutrients. Interestingly, apart from prey items, many living organisms have been observed in these bladder traps^21,22^. Indeed, Charles Darwin reported “In all cases, the bladders with decayed remains swarmed with living Algae of many kinds, Infusoria, and other low organisms, which evidently lived as intruders”^23^, wherein “Infusoria” represents a collective term for aquatic microorganisms and primarily ciliate protozoans. Recent studies indicate that a diverse microbial community exists within *Utricularia* bladder traps, with cohabiting ciliates perhaps representing commensals feeding on this community or on decaying remains^24–26^. However, the abundance and diversity of ciliates living in *Utricularia* bladder traps remains to be fully established.

In this partial survey, we report a total of 19 different putative *Tetrahymena* species and two closely related *Glaucoma* lineages from Taiwan, 13 of which are new species. These *Tetrahymena* species were recovered from bladder traps of seven different species of *Utricularia* plants, indicating a previously unknown but significant symbiotic relationship between *Utricularia* plants and *Tetrahymena* ciliates in natural environments. We propose that *Tetrahymena* has adopted a specialized feeding behavior that involved adapting to the commensal microbial community in *Utricularia* bladder traps, possibly allowing it to play a critical role as a scavenger in this particular environment. This scavenging behavior is evidenced by the capture of large numbers of *Tetrahymena* using an artificial trapping method. Our survey has also suggested that *Tetrahymena* species populations from a single pond are dynamic, and that the biodiversity of *Tetrahymena* species is much greater than currently acknowledged.

## Results

### *Tetrahymena* species inhabit *Utricularia* bladder traps

Initially, we collected two species of *Utricularia* (*U*. *aurea* and *U*. *gibba*) from a small abandoned agricultural irrigation pond located in Taipei, Taiwan (25°07′41.1′′N 121°38′09.8′′E). We observed ciliate-like microorganisms actively swimming inside the bladder traps of these plants (Supplementary Movie 1). We examined the bladder traps of *U*. *aurea* and found that about 90% of them harbored *Tetrahymena*-like ciliates. Optical microscopy revealed these unknown ciliates as being morphologically similar to *Tetrahymena thermophila*, exhibiting its characteristic pear-shaped body and swimming by undulating numerous cilia. DAPI staining of the nuclei revealed nuclear dimorphism, with some individuals possessing two types of cell nuclei (a micronucleus and a macronucleus, which is a unique feature of ciliates) and others only having a macronucleus (which may represent amicronucleate *Tetrahymena* species)^27^.

**Figure 1:**
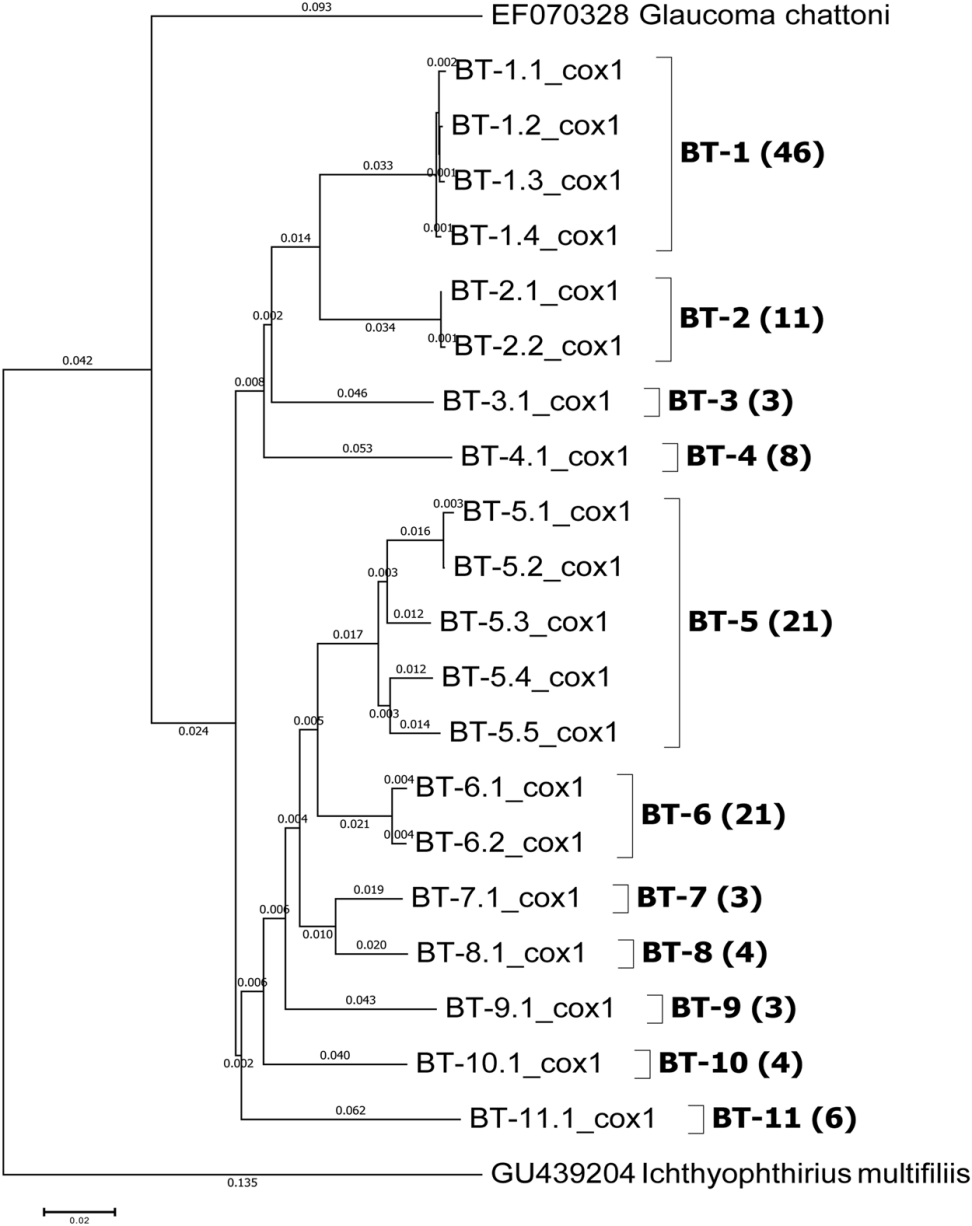
Classification of *Tetrahymena* ciliates isolated from *Utricularia* bladder traps. Phylogenetic analysis to classify and group species of *Tetrahymena* ciliate isolated from *Utricularia* bladder traps. The analysis involved 22 *COX1* sequences, including 20 from samples collected from the wild. *Ichthyophthirius multifiliis* was used as an outgroup and *Glaucoma chattoni* was used as a closely-related species. All ambiguous positions were removed from each sequence pair, resulting in a dataset of 1030 nucleotide positions. The Neighbor-Joining tree is drawn to scale, with branch lengths (shown on branches) in the same units as evolutionary distances based on *COX1* sequences. Numbers of *Tetrahymena* isolates for each lineage are shown in brackets. Scale bar: the number of base substitutions per site.

**Figure 2:**
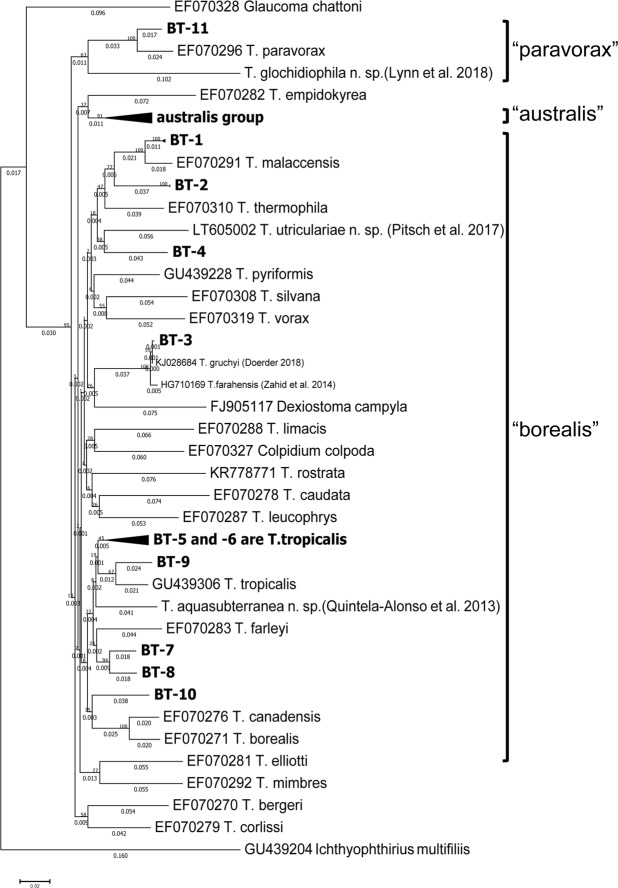
Phylogenetic analysis of *Tetrahymena* species isolated from bladder traps and named *Tetrahymena* species. Phylogenetic analysis of 67 *Tetrahymena* isolates based on *COX1* gene sequences, including 20 wild isolates grouped into 11 lineages (BT1~11), 43 named *Tetrahymena* species, three closely-related species (*Glaucoma chattoni*, *Dexiostoma campyla* and *Colpidium colpoda*), and *Ichthyophthirius multifiliis* as an outgroup. The *COX1* sequences are listed in Supplementary Table 2. The three main clades of *Tetrahymena* are shown on the right. All ambiguous positions were removed from each sequence pair, resulting in a dataset of 768 nucleotide positions. The Neighbor-Joining tree is drawn to scale, with branch lengths (shown on branches) in the same units as evolutionary distances based on *COX1* sequences. Scale bar: the number of base substitutions per site.

To better identify the species of ciliate, we employed *COX1* barcoding. We adopted the criteria of *Tetrahymena* species that strains exhibiting <1% *COX1* sequence divergence are considered the same species and those with >4% sequence divergence to be different species^13,27^. Among the 18 clones analyzed, we obtained 18 *COX1* sequences that could be grouped into two clusters that diverged by about 10.5%, suggesting that they represent two different species. We performed a BLAST search against known *COX1* sequences in the NCBI GenBank database and the most similar sequence to ours was *COX1* accession GU439296 from *Tetrahymena thermophila*. However, our sequence (BT-4 in Figs 1 and 2) still diverges from that accession by about 9%, indicating that our sequence is from a species not represented in the database and is potentially a new species. Our other sequence (BT-1 in Figs 1 and 2) had a closest match to *COX1* accession EF070291 of *Tetrahymena malaccensis*, but still exhibited a sequence difference of about 3%, indicating that it may potentially be a new species.

This preliminary assessment prompted us to expand the survey and collect more *Utricularia* from around Taiwan, the distribution of which has been previously reported^28,29^.

### *Tetrahymena* is abundant in *Utricularia* bladder traps

**Figure 3:**
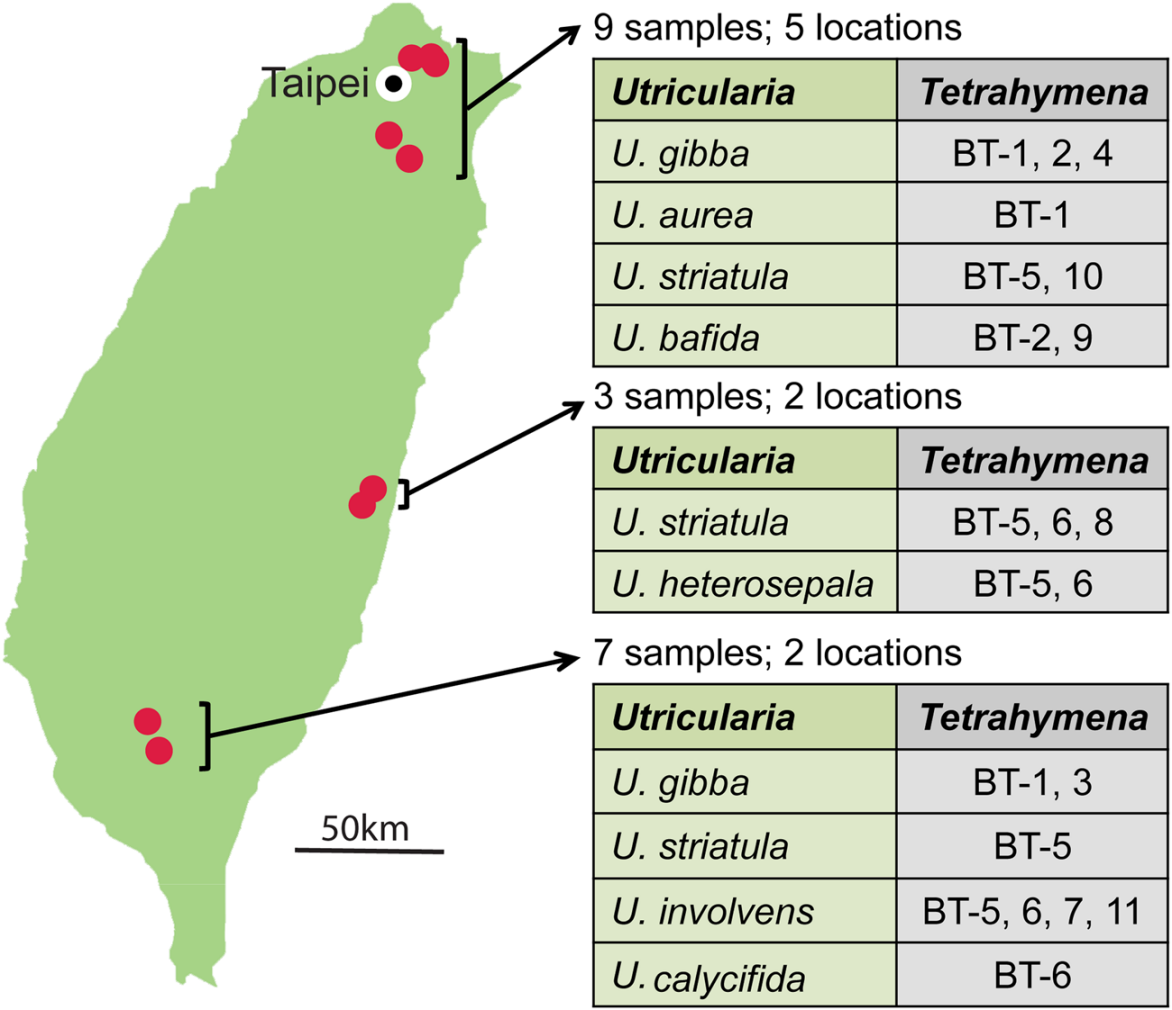
Sampling locations for *Utricularia* plants in Taiwan. The map of Taiwan shows the sampling locations (red dots) for *Utricularia* plant samples. Tables at right show which *Tetrahymena* species were isolated from which species of *Utricularia*.

Our extended *Utricularia* survey yielded 19 samples from nine locations in the north, south, and east of Taiwan, and included seven different species of *Utricularia* (Table 1 and Fig. 3). We collected 130 *Tetrahymena*-like ciliate clones from 15 of the 19 *Utricularia* samples. *COX1* barcoding revealed that all these isolates belonged to the genus *Tetrahymena* (Fig. 2), with the 130 isolates yielding 20 different *COX1* sequences that could be grouped into 11 different putative species based on phylogenetic analysis (Fig. 1). We have named these putative species BT-1 to 11 to represent that the *Tetrahymena* species had been collected from the bladder traps of *Utricularia* plants. Any given species may be represented by multiple *COX1* haplotypes in the population, but most of these haplotypes are likely to differ by less than 1%. However, BT-5 exhibited particularly pronounced haplotype diversity in which the difference was much larger than 1% for most isolates, thus they could also be considered as potential new species (Fig. 1). We downloaded all available *Tetrahymena COX1* sequences which represent the known named species from the GenBank database to generate a phylogenetic tree incorporating all of our new *Tetrahymena* sequences. This analysis revealed that BT-11 belongs to the “paravorax” clade, whereas all others belong to the “borealis” clade, and none of our sequences could be identified as belonging to the “australis” clade (Fig. 2)^7,13,30,31^. This analysis also allowed us to establish that BT-3 is either *T*. *gruchyi* or *T*. *farahensis* and that BT-5 and BT-6 are *T*. *tropicalis* (an ambiguous taxon exhibiting ~6.2% intraspecific *COX1* sequence divergence among many isolates listed in the GenBank database). BT-1 is potentially a new species closely related to *T*. *malaccensis*, from which it has a *COX1* sequence divergence of ~3%, and our other seven new sequences likely represent new species. These results indicate that large numbers of diverse and hitherto unknown *Tetrahymena* species inhabit *Utricularia* bladder traps and our data confirm the significant relationship between *Utricularia* plants and *Tetrahymena* ciliates in natural environments.

**Table 1:** *Tetrahymena* species isolated from bladder traps of various *Utricularia* species.

*Utricularia* species	*Tetrahymena* species BT
*U*. *gibba* (5)	1, 2, 3, 4
*U*. *striatula* (4)	5, 6, 8, 10
*U*. *aurea* (2)	1,4
*U*. *bafida* (1)	2, 9
*U*. *involvens* (1)	5, 6, 7, 11
*U*. *calycifida* (1)	6
*U*. *heterosepala* (1)	5, 6

Table showing the species of *Utricularia* plants collected in this study (number of specimens in brackets) and the corresponding lineages of *Tetrahymena* isolated from their bladder traps.

### Biogeography of *Tetrahymena* species in Taiwan

Our partial survey provides a preliminary picture of the biogeographic distribution of *Tetrahymena* species in Taiwan, based on *Utricularia* sampling sites (Fig. 3). The genus *Tetrahymena* is globally distributed, but there is strong evidence for population structuring in *T*. *thermophila*^32,33^. Among our collection of *Tetrahymena* specimens, the BT-1, BT-5 and BT-6 lineages are relatively widespread in the northern and southern areas of Taiwan. In contrast, the BT-2, BT-4, BT-9 and BT-10 lineages are restricted to the north of the island, BT-3, BT-7 and BT-11 occur only in the south, and BT-8 is an eastern lineage.

### The symbiotic relationship of *Tetrahymena* and *Utricularia*

To better understand the relationship between *Tetrahymena* and *Utricularia*, we further assessed the habitats from which our eleven *Tetrahymena* lineages had been collected (Table 1). Our results show that multiple *Tetrahymena* species can be found in the same *Utricularia* species. For example, we identified four different species from *U*. *gibba* (BT-1, BT-2, BT-3 and BT-4). Moreover, different species of *Utricularia* can harbor the same *Tetrahymena* species. For instance, BT-1 was isolated from both *U*. *gibba* and *U*. *aurea* plants. These findings indicate a lack of species specificity in their interrelationship, but we cannot rule out the possibility that certain *Tetrahymena* species may be better adapted or exhibit a preference for particular species of *Utricularia*. Thus, the relationship between *Tetrahymena* and *Utricularia* may be best described as facultative symbiosis. The highly abundant and diverse *Tetrahymena* species we found living in *Utricularia* bladder traps may also imply that they possess unique physiological features that facilitates this symbiosis. Moreover, observations of the wild samples suggested that these *Tetrahymena* may feed on the decaying animal prey in the bladder traps (Supplementary Movies 2 and 3). We also observed that cultured *Tetrahymena* tended to attach strongly to animal tissue fibers present in the culture medium (Supplementary Movie 4). Indeed wild-caught specimens of *Tetrahymena* displayed remarkable adaptation to growing in our axenic culture medium that mainly comprised proteose-peptone of animal tissue origin. These observations suggest that *Tetrahymena* may exhibit scavenging behavior.

To assess this possibility further, we designed a simple “artificial trap” containing a piece of animal tissue as bait to attract *Tetrahymena* ciliates. We placed our trap in the open water of a pond for ~16 hours and then examined in the laboratory which organisms had been attracted into the chamber. In total, we examined eight samples (six from the pond located at 25°02′32.2′′N 121°36′46.3′′E; the other two from 25°02′43.0′′N 121°36′48.5′′E and 25°07′41.1′′N 121°38′09.8′′E, respectively.), all of which harbored *Tetrahymena*-like ciliates and yielded 185 isolated clones. Based on our *COX1* barcoding protocol, these 185 clones represent 30 different *COX1* haplotypes that could be grouped into 13 different putative species (Supplementary Fig. 1). We have named these putative species AT-1 to 13 to represent that they were collected using our artificial trapping method. Phylogenetic analysis revealed that AT-7 and AT-8 clustered in the “paravorax” clade and that AT-3 and AT-6 grouped in the “australis” clade. The remaining lineages lay in the “borealis” clade, except for two divergent species, AT-12 and AT-13, that grouped with the genus *Glaucoma* (Supplementary Fig. 2). AT-1 could be identified as either *T*. *gruchyi* or *T*. *farahensis*. AT-2 (which is the same as BT-1 species by the criteria of <1% *COX1* sequence divergence) is potentially a new species closely related to *T*. *malaccensis*, from which its *COX1* sequence diverged by ~3%. AT-3, AT-4 and AT-6 appear to represent *T*. *shanghaiensis*, *T*. *pyriformis* and *T*. *alphapoecilia SIN*, respectively, and the remaining eight lineages may be new species. These results indicate that an abundant and diverse *Tetrahymena* community exists in the pond, and that artificial trapping is an efficient collection technique, as alluded to in a previous report^8^.

### Different natural habitats harbor divergent *Tetrahymena* communities

**Figure 4:**
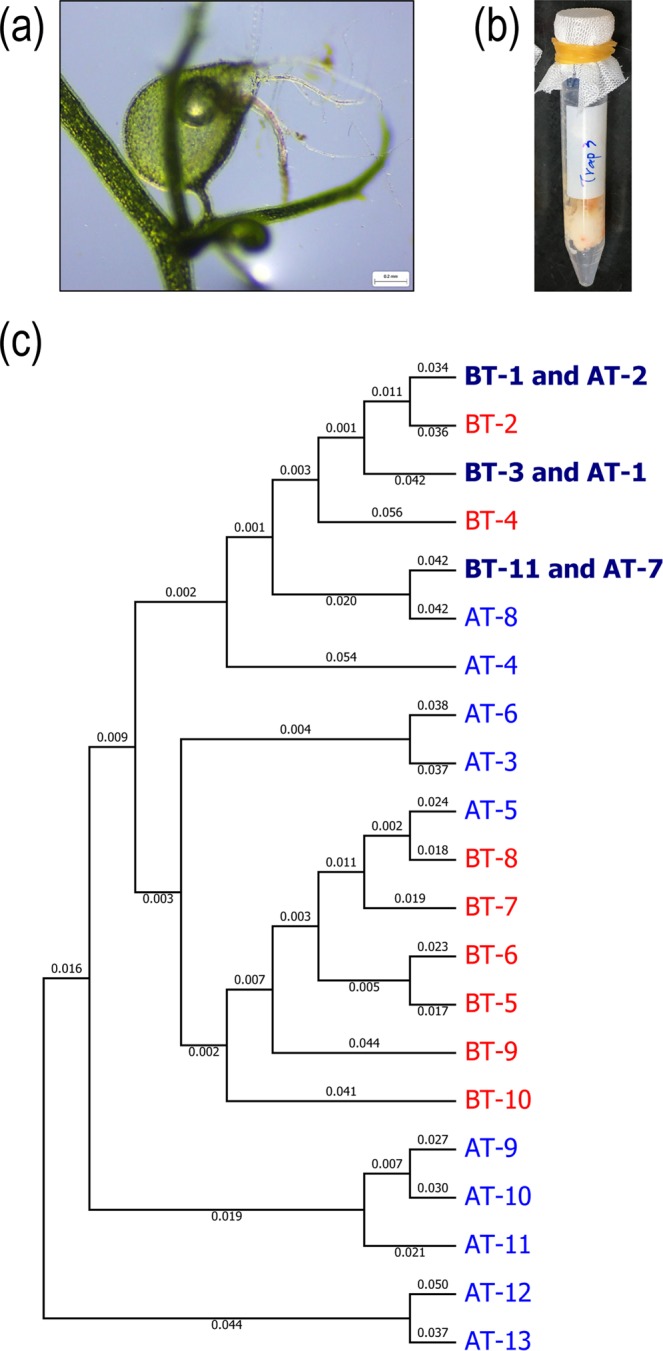
Comparison of *Tetrahymena* species collected from *Utricularia* bladder traps and by artificial trapping. (**a**) The image shows a bladder trap of *Utricularia gibba*. (**b**) The image shows an artificial trap. (**c**) The topology of the phylogenetic tree for *Tetrahymena* species was generated by the Neighbor-Joining algorithm based on *COX1* gene sequences. All ambiguous positions were removed for each sequence pair, resulting in a dataset of 994 nucleotide positions. Red labels show species only isolated from *Utricularia* bladder traps. Pale blue labels identify species only isolated by the artificial trapping method. Dark blue labels represent overlapping species, i.e., isolated from both collection methods.

When we compared the *Tetrahymena* species collected from our two natural habitats, symbiotes in bladder traps and artificially trapped free-living ciliates in the open water of a pond, we found that the communities only overlapped marginally. Of the 21 distinct lineages identified using both approaches, only three had been isolated using both methods: BT-1 and AT-2, BT-3 and AT-1, BT-11 and AT-7 (each pair share a similar *COX1* sequence of < 1% differences) (Fig. 4). This outcome suggests that distinct lineages of *Tetrahymena* may inhabit various natural environments. This supposition is supported by the fact that we have identified seven different *Tetrahymena* species from three types of natural habitats in and around one pond in Taipei, Taiwan (25°07′41.1′′N 121°38′09.8′′E); two inhabited *Utricularia* bladder traps, four were found in the open water, and one was isolated from a small pool of water in a tree crevice (Supplementary Fig. 3).

### Sympatric *Tetrahymena* species populations dynamically change within a pond

**Figure 5:**
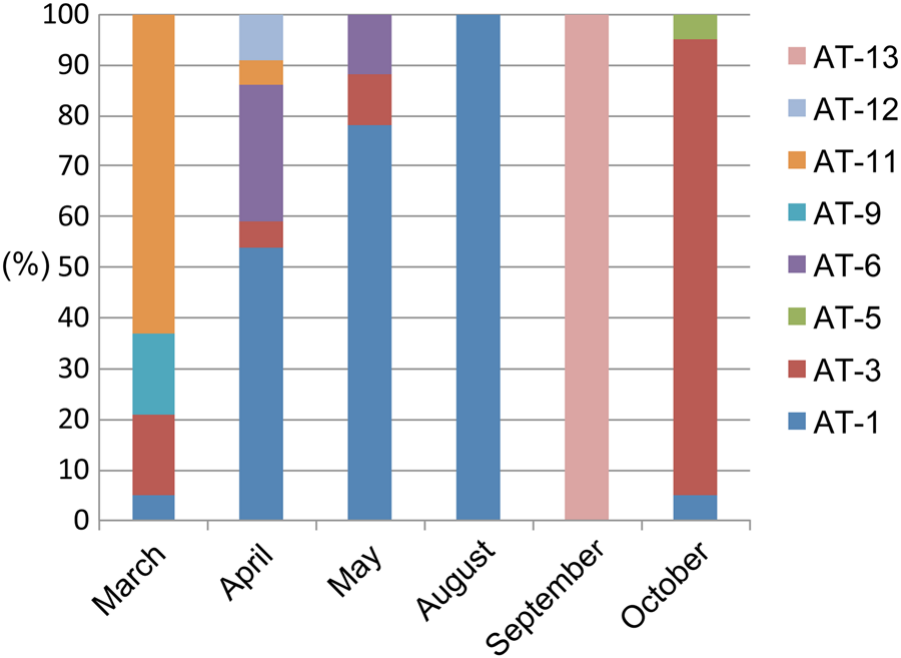
Dynamic changes in a *Tetrahymena* community sampled from a single location in a pond. Relative abundances (%, y-axis) of *Tetrahymena* trapped in different months (March, April, May, August, September and October of 2018) from a single pond in Academic Sinica, Taipei, Taiwan.

To better understand the dynamics of *Tetrahymena* populations at a single location, we repeatedly sampled *Tetrahymena* species using our artificial trapping method at a single pond in the grounds of Academia Sinica, Taipei, Taiwan (25°02′32.2′′N 121°36′46.3′′E). We collected six samples over a period of eight months, i.e. in March, April, May, August, September and October of 2018. We recovered four *Tetrahymena* species (AT-1, AT-3, AT-9 and AT-11) in the March sample, five species (AT-1, AT-3, AT-6, AT-11 and AT-12) in the April sample, three species (AT-1, AT-3 and AT-6) in the May sample, one species (AT-1) in the August sample, one species (AT-13) in the September sample, and three species (AT1, AT-3 and AT-5) in the October sample (Fig. 5). This preliminary survey showed that up to six different *Tetrahymena* species and two different *Glaucoma* species were found at a single location in the pond, but species combinations varied throughout the year. Together, our findings indicate that the number of *Tetrahymena* species could be greatly underestimated by single field surveys at a sole location, and imply that global diversity of *Tetrahymena* species may be much greater than currently acknowledged.

## Discussion

In this study, we investigated pond habitat and *Utricularia* plant bladder traps and found a diversity of *Tetrahymena* ciliates. We consider the interrelationship between *Tetrahymena* and *Utricularia* to be a facultative symbiosis. *Tetrahymena* appears to be a commensal organism in the microbial community of *Utricularia* bladder traps. It can directly feed on axenic culture medium made from animal tissues, and an artificial trap with decaying animal tissue bait attracted *Tetrahymena* ciliates. Thus, we conclude that *Tetrahymena* species exhibit strong scavenging behavior, indicating that the ecological role of *Tetrahymena* might be as “a scavenger”, assisting in the process of animal tissue decomposition in the food web and facilitating energy recycling in the ecosystem. It would be interesting to investigate which physiological features contribute to *Tetrahymena*’s scavenging behavior. For instance, *Tetrahymena* possesses a secretory organelle called the “mucocyst” that harbors proteases, and it has been linked to responses to changing environments^34,35^.

Aside from North America, previous surveys of *Tetrahymena* species have been rather limited. Some species have been collected from parts of Asia, but none from Taiwan. This study represents the first on the genus *Tetrahymena* in Taiwan, a subtropical East Asian island. We report 19 *Tetrahymena* species and two closely related *Glaucoma* lineages, 13 of which may be new species. This diversity in natural environments suggests that the island may harbor a rich biodiversity for this protist. Broader surveys will be necessary to establish the biodiversity of Taiwanese *Tetrahymena*, and expanding the survey to other islands in the region will likely prove fruitful.

In this study, we found that only two among the 19 *Tetrahymena* species identified belonged to the “australis” clade, which was considered to be less diversified with closely related species than the “borealis” clade^7,13^. This could represent an interesting biogeographical or habitant specialization. However, it is also possible that our collection methods are biased toward one clade. These issues can be addressed by altering collecting methods and performing a wider range of field survey.

Little is known about the dynamics of *Tetrahymena* ciliate populations in nature and what impacts they have on ecosystems. In this study, we identified a diverse *Tetrahymena* community inhabiting a single location in a pond, indicating that large numbers of sympatric species can live together. This result also indicates that *Tetrahymena* populations may actively compete for resources in the wild. Further investigations will be necessary to assess the interplay among different *Tetrahymena* populations and reveal their ecological roles in the wild. Moreover, we found *Tetrahymena* communities to be dynamic, perhaps changing in response to the environment. It would be very informative to develop long-term monitoring to track ciliate communities over time and reveal which factors (such as location, weather, season or human activity) impact *Tetrahymena* population dynamics in nature.

## Methods

### *Utricularia* plant collection and *Tetrahymena*-like ciliate examination

*Utricularia* plant samples were collected from different locations in Taiwan that are listed in Supplementary Table 1. A simple examination was conducted to verify whether *Tetrahymena*-like ciliates exist in the bladder traps of *Utricularia* plants. We dissected and isolated individual bladder traps from the stem of *Utricularia* plants and placed them separately in the wells of 96- well plate containing standard proteose-peptone culture medium for *T*. *thermophila*. The existing *Tetrahymena*-like ciliates would be cultivated in the well if they were released from the bladder traps. The medium drop plate can replace the 96-well plate for better observations and manipulations under a dissecting microscope.

### *Tetrahymena* ciliate isolation

Based on the standard protocol for inbreeding *T*. *thermophila* strains used in the laboratory, wild collecting ciliates were cultivated by standard SPP or Neff medium and kept to grow at room temperature in a moist incubator. We conducted single-cell isolation from the cultured *Tetrahymena*-like ciliates, which were isolated from *Utricularia* bladder traps, artificial trapping in the pond, and various habitat samples in which the microbial communities could contain multiple species. Isolated single-cell was cultivated in a small drop (~20–30 μl) of Neff medium for several days. The pure *Tetrahymena* clones were established and generated when they adapted to Neff medium. Each established pure clone was prepared to extract genomic DNA when the cultured cell density reached ~2 × 10^5^ cells/ml.

### The artificial trapping method

A simple “artificial trap” is designed as a chamber (a 15 ml plastic FALCON tube) containing a piece of animal meat (we used a piece of shrimp meat about 0.5 cubic centimeters in size for all sample collections) as bait to attract *Tetrahymena* ciliates. The opening to the chamber was covered with a filter (Nylon cloth, the hole size is about 0.5 mm) to prevent larger carnivores from entering it. We placed the “artificial trap” device in shallow water areas, approximately 10–20 cm deep at the bottom of the pond for ~16 hours. After retrieving to the laboratory, immediately the fluid of the trapping chamber was poured into cell culture plates and the antibiotics and antifungals were added to prevent the over-growth of bacteria and fungi. The cultured *Tetrahymena*-like ciliates usually became readily detectable in 1–3 days.

### *COX1* barcoding and phylogenetic relationship analysis

*Tetrahymena* genomic DNA were extracted by a QIAGEN commercial kit and used as templates to perform polymerase chain reactions (PCR) to amplify the partial *COX1* gene using the primer set, the forward primer COI-FW 5′-ATGTGAGTTGATTTTATAGAGCAGA-3′^12^ and the reverse primer FolB 5′-TAAACTTCAGGGTGACCAAAAAATCA-3′^36^. The PCR products were purified and sequenced to obtain about 1 kb length of a partial *COX1* sequence. The phylogenetic analysis was conducted by using the Neighbor-Joining method^37^. The percentage of replicate trees in which the associated taxa clustered together in the bootstrap test (1000 replicates) are shown next to the branches^38^. The tree was drawn to scale, with branch lengths (next to the branches) in the same units as those of the evolutionary distances used to infer the phylogenetic tree. The evolutionary distances were computed using the Kimura 2-parameter method^39^ and were in the units of the number of base substitutions per site. All ambiguous positions were removed for each sequence pair. Evolutionary analyses were conducted in MEGA7^40^.

## Supplementary information

Supplementary Movie 1

Supplementary Movie 2

Supplementary Movie 3

Supplementary Movie 4

Supplementary Information

Supplementary Table 1

Supplementary Table 2
