# Supplementary material for: Abundant and diverse Tetrahymena species living in the bladder traps of aquatic carnivorous Utricularia plants
Source: Sci Rep. 2019 Sep 20;9:13669. doi: 10.1038/s41598-019-50123-1 (PMC6754427; doi:10.1038/s41598-019-50123-1)

Abundant and diverse Tetrahymena species living in the bladder traps of aquatic carnivorous Utricularia plants

Chao-Yin Cheng, Shang-Lin Chang, I-Ting Lin, & Meng-Chao Yao

Supplementary Figure 1. Classification of *Tetrahymena* ciliates isolated from the artificial trapping method. Phylogenetic analysis to classify and group species of *Tetrahymena* ciliate isolated from artificial trapping. The analysis involved 32 *COX1* sequences, including 30 from samples collected from the wild. *Ichthyophthirius multifiliis* was used as an outgroup and *Glaucoma chattoni* was used as a closely-related species. All ambiguous positions were removed for each sequence pair, resulting in a dataset of 996 nucleotide positions. The Neighbor-Joining tree is drawn to scale**,** with branch lengths (shown on branches) in the same units as evolutionary distances based on *COX1* sequences. Numbers of *Tetrahymena* isolates for each lineage are shown in brackets. Scale bar: the number of base substitutions per site.

Supplementary Figure 2. Phylogenetic analysis of *Tetrahymena* species isolated from artificial trapping and named *Tetrahymena* species*.* Phylogenetic analysis of 77 *Tetrahymena* isolates based on *COX1* gene sequences, including 30 wild isolates grouped into 13 lineages (AT1~13), 43 named *Tetrahymena* species, three closely-related species (*Glaucoma chattoni*, *Dexiostoma campyla* and *Colpidium colpoda*), and *Ichthyophthirius multifiliis* as an outgroup. The *COX1* sequences are listed in Supplementary Table 2. The three

main clades of *Tetrahymena* are shown on the right. All ambiguous positions were removed for each sequence pair, resulting in a dataset of 810 nucleotide positions. The Neighbor-Joining tree is drawn to scale**,** with branch lengths (shown on branches) in the same units as evolutionary distances based on *COX1* sequences. Scale bar: the number of base substitutions per site.

Supplementary Figure 3. Phylogenetic analysis of *Tetrahymena* species isolated from three types of natural habitats in and around one pond in Taipei, Taiwan (25°07'41.1"N 121°38'09.8"E)*.* Phylogenetic analysis of different types of natural inhabiting *Tetrahymena* lineages based on *COX1* gene sequences, including that red labels show two *COX1* haplotypes from *Utricularia* bladder traps, pale blue labels show five by artificial trapping from the open water, and green labels show two from a small pool of water in tree holes. All ambiguous positions were removed for each sequence pair, resulting in a dataset of 997 nucleotide positions. The Neighbor-Joining tree is drawn to scale**,** with branch lengths (shown on branches) in the same units as evolutionary distances based on *COX1* sequences. Scale bar: the number of base substitutions per site.

Supplementary Table 1. The list of collecting *Tetrahymena* isolated from *Utricularia* bladder traps. Table showing the information of the field survey of isolating *Tetrahymena* isolates including the isolate number, the collection date, collection site (GPS coordinate) and the species of *Utricularia* plants, etc.

Supplementary Table 2. The list of the *COX1* sequences used in the phylogenetic analysis. Table showing the *COX1* accession number in the GenBank database and the *COX1* sequences that were obtained in this study.

Supplementary Movie 1. *Tetrahymena*-like ciliates swim inside the bladder trap of *Utricularia aurea*.

Supplementary Movie 2 and 3. *Tetrahymena*-like ciliates associate with the decaying animal prey trapped by carnivorous *Utricularia* plants.

Supplementary Movie 4. Feeding behavior of *Tetrahymena* present in the culture medium containing animal tissue fibers.

Supplementary Fig. 1 **Cheng_Supp_Fig1**

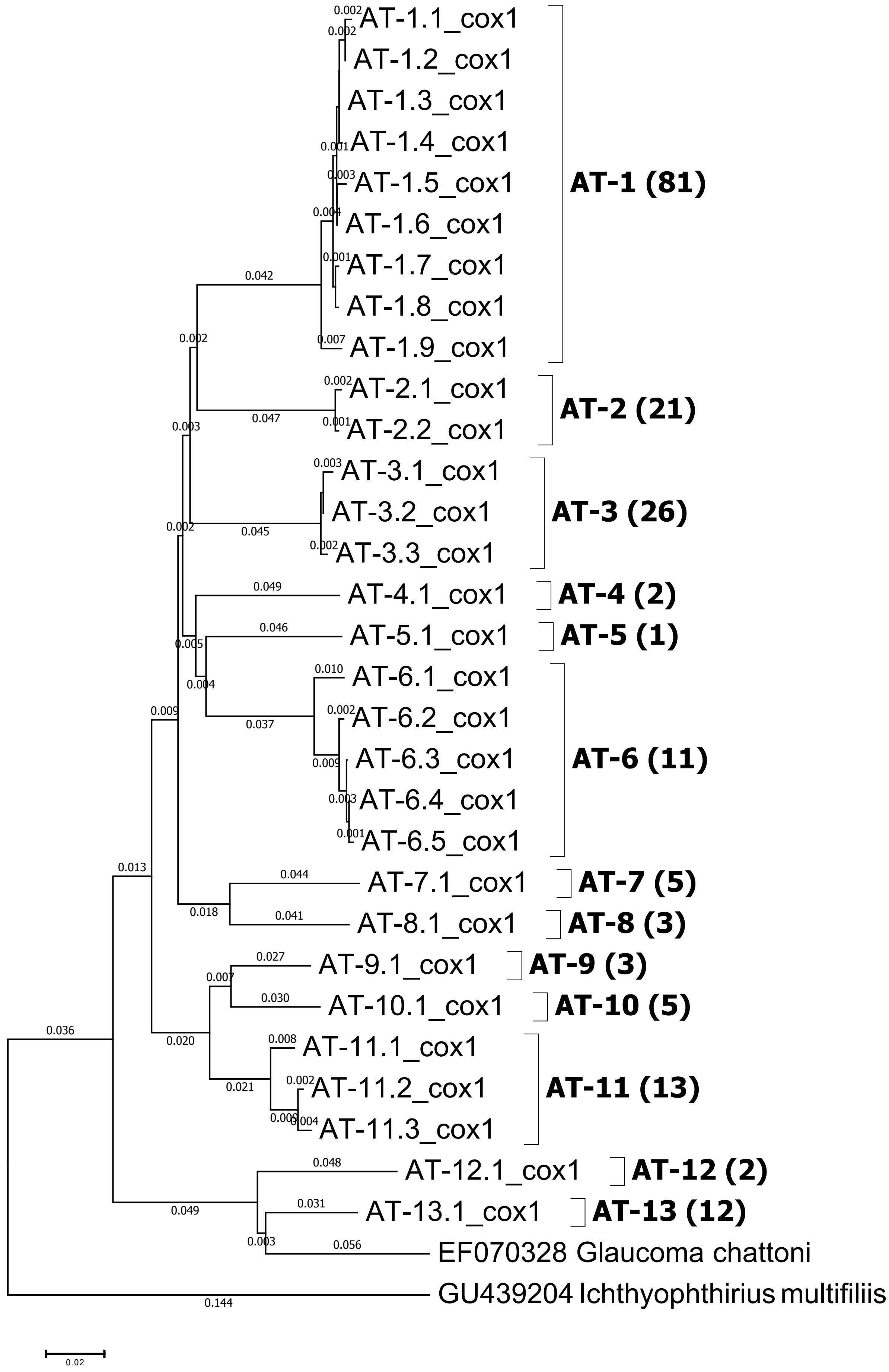

Supplementary Fig. 2

Cheng_Supp_Fig2

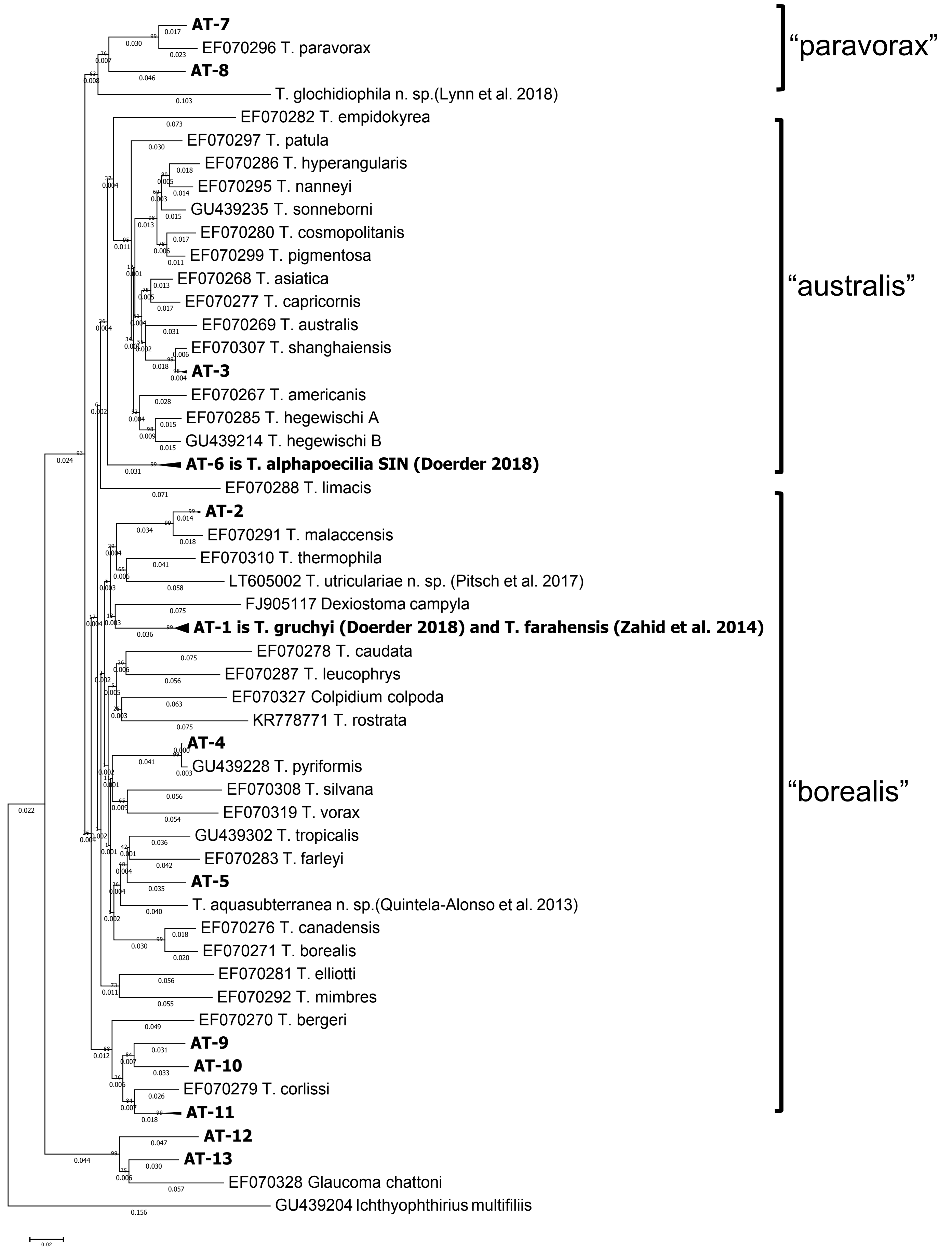

Supplementary Fig. 3

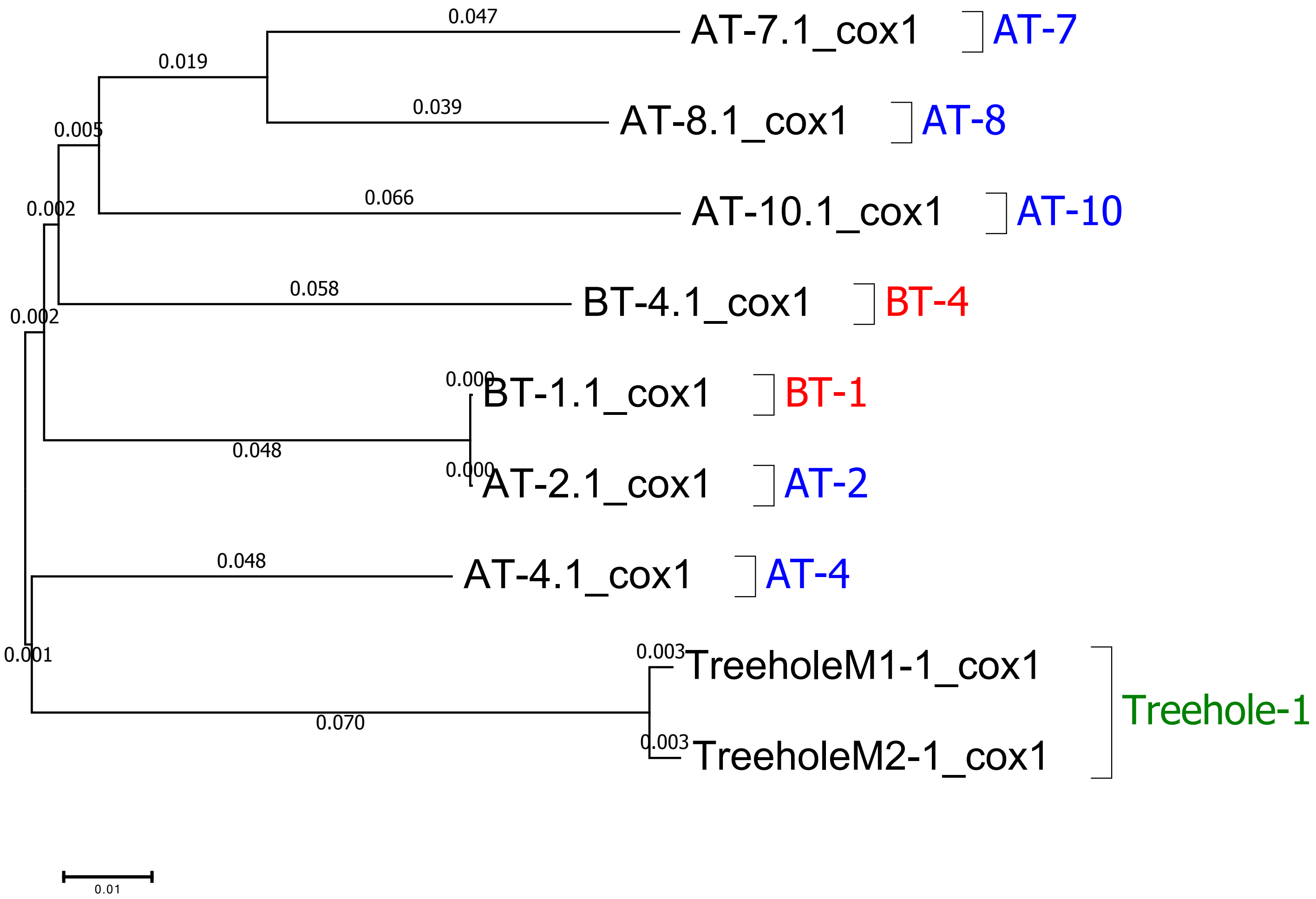

Supplement: Supplementary file 5 — Supplementary Information [file 41598_2019_50123_MOESM5_ESM.pdf]
